# Developing a Portable Autofluorescence Detection System and Its Application in Biological Samples

**DOI:** 10.3390/s24113351

**Published:** 2024-05-23

**Authors:** Jiaxing Zhou, Yunfei Li, Jinfeng Zhang, Fuhong Cai

**Affiliations:** School of Biomedical Engineering, Hainan University, Sanya 572000, China; qq2628783938@outlook.com (J.Z.); 1457893655@foxmail.com (J.Z.); caifuhong@hainanu.edu.cn (F.C.)

**Keywords:** fluorescence monitoring, advanced glycation end-products (AGEs), portable detection system, diabetes management

## Abstract

Advanced glycation end-products (AGEs) are complex compounds closely associated with several chronic diseases, especially diabetes mellitus (DM). Current methods for detecting AGEs are not suitable for screening large populations, or for long-term monitoring. This paper introduces a portable autofluorescence detection system that measures the concentration of AGEs in the skin based on the fluorescence characteristics of AGEs in biological tissues. The system employs a 395 nm laser LED to excite the fluorescence of AGEs, and uses a photodetector to capture the fluorescence intensity. A model correlating fluorescence intensity with AGEs concentration facilitates the detection of AGEs levels. To account for the variation in optical properties of different individuals’ skin, the system includes a 520 nm light source for calibration. The system features a compact design, measuring only 60 mm × 50 mm × 20 mm, and is equipped with a miniature STM32 module for control and a battery for extended operation, making it easy for subjects to wear. To validate the system’s effectiveness, it was tested on 14 volunteers to examine the correlation between AGEs and glycated hemoglobin, revealing a correlation coefficient of 0.49. Additionally, long-term monitoring of AGEs’ fluorescence and blood sugar levels showed a correlation trend exceeding 0.95, indicating that AGEs reflect changes in blood sugar levels to some extent. Further, by constructing a multivariate predictive model, the study also found that AGEs levels are correlated with age, BMI, gender, and a physical activity index, providing new insights for predicting AGEs content and blood sugar levels. This research supports the early diagnosis and treatment of chronic diseases such as diabetes, and offers a potentially useful tool for future clinical applications.

## 1. Introduction

Advanced glycation end-products (AGEs) are complex molecules formed through non-enzymatic reactions [1]. They can be ingested from food, as well as produced endogenously, gradually accumulating within the human body [2]. The accumulation of advanced glycation end-products (AGEs) occurs in the progression of various chronic diseases, including diabetes, renal diseases, cardiovascular diseases, and neurological disorders [3,4,5]. For instance, research on AGEs has uncovered their pivotal role in accelerating the development of complications associated with diabetes [6,7]. This influence extends beyond mere hyperglycemia to encompass profound damage at the microscopic molecular level [8,9]. Investigating the dynamics of advanced glycation end-products (AGEs) in the human body is essential for the early detection and intervention of chronic diseases [10]. For example, diabetes mellitus presents a significant and increasing challenge globally, affecting both developed and developing countries. As of 2021, approximately 537 million adults worldwide are living with diabetes. This number is projected to increase to 643 million by 2030 and 783 million by 2045 [11]. It is noteworthy that a substantial proportion of diabetes cases remain undiagnosed, with about half of the individuals unaware of their condition [12,13]. Although diabetes can be managed through medication, effective treatment necessitates early detection. Unfortunately, diagnosis often occurs several years after the onset of diabetes, typically at the commencement of complications [13].

AGEs are implicated in numerous chronic diseases through two primary formation pathways: exogenous and endogenous [2]. Exogenous AGEs accumulate from environmental exposures such as cigarette smoke, consumption of highly processed foods, particularly those processed with heat, and sedentary lifestyles, all of which contribute to increased formation rates of these harmful compounds [5,6]. Conversely, endogenous AGEs originate internally under conditions of elevated blood sugar, where reducing sugars like glucose, lactose, and fructose undergo non-enzymatic glycation reactions with proteins and lipids [14]. This internal process is known as the Maillard reaction, a crucial pathway for AGEs formation within the body.

AGEs are present in human blood and tissues, and their detection relies on drawing blood [15] or obtaining bodily fluids [16] for biochemical analysis [17]. This biochemical testing is not only costly, but is also time-consuming, requiring 8–9 h. Developing non-invasive, convenient, and pain-free methods of detection is essential for the practical continuous measurement of advanced glycation end-products (AGEs) in human blood and tissues, serving as a foundation for long-term monitoring and research into their role in chronic diseases. Over the past decades, a multitude of technologies and corresponding equipment have been developed to pursue more convenient and cost-effective detection methods [18,19,20]. Within this domain, numerous optical spectroscopic methods have attracted considerable attention due to their potential in enhancing analytical precision and reducing operational complexities [21,22,23,24]. The optical non-destructive testing method is implemented by analyzing the autofluorescence characteristics of AGEs, utilizing the unique fluorescence properties of molecules to specifically detect target molecules. The primary absorption band of advanced glycation end-products (AGEs) lies between 340 nm and 420 nm, with the fluorescence spectrum spanning the 420 nm to 600 nm range [3]. Instruments designed for detecting autofluorescence have been developed and validated through small-scale clinical trials targeting human AGEs. Researchers utilize fiber optic spectrometers for detection, employing an excitation light source ranging from 300 to 400 nm to irradiate the skin and capture signals with a glass fiber optic spectrometer. However, transdermal detection is hindered by the low efficiency of spontaneous fluorescence. In vivo, where the concentration of AGEs is relatively low, the fluorescent peaks of AGEs are significantly diminished by the skin’s strong and time-varying spontaneous fluorescence, further compounded by associated shot noise, rendering the AGEs’ fluorescence even weaker. This presents substantial challenges in constructing reliable predictive models for AGEs concentration. Furthermore, the propagation characteristics of fluorescence signals are influenced by the skin’s color composition, contact pressure, and the optical parameters of biological tissues. Researchers have attempted to circumvent the influence of skin color by analyzing tissues such as the retina and wrist, but the fragile structure of the retina is not conducive to long-term monitoring. Correcting AGEs’ fluorescence signals through calibration models requires prior information such as skin type, age, and the subject’s habits, among others. The introduction of additional information necessitates preliminary collection, which is not favorable for reducing detection costs. Building on the fluorescence properties of advanced glycation end-products (AGEs), this paper introduces a novel portable skin fluorescence detection device designed for the assessment of AGEs. The device comprises a control module, an LED emission module, and a detection module. The LED emission module emits a 395 nm wavelength laser to excite the AGEs in the skin, causing them to fluoresce. This fluorescence is then captured by a photodiode within the detection module, enabling the measurement of AGEs concentration based on the intensity of the fluorescence signal. To account for the impact of tissue optical parameters on the fluorescence signal, a 520 nm LED is utilized to measure skin reflectance, facilitating the correction of AGEs’ fluorescence values. The device is equipped with a miniaturized STM32 microcontroller and a 3.7 v battery, serving as both the control and power modules, which simplifies the process of long-term monitoring of AGEs’ fluorescence intensity. To validate the effectiveness of this system, a study was conducted with 14 volunteers to analyze the correlation between AGEs and glycated hemoglobin levels. Moreover, the device was used to perform long-term monitoring of AGEs’ fluorescence and blood glucose levels in volunteers, revealing a correlation exceeding 0.95 between the two, indicating that AGEs levels reflect changes in blood glucose levels to a significant extent. Furthermore, by developing a multivariate prediction model, the study also found that AGEs levels are associated with age, BMI, gender, and a physical activity index, providing new insights into predicting AGEs content and blood glucose levels. This integration of portable technology with fluorescence measurement offers a promising tool for the non-invasive and continuous monitoring of biomarkers that are critical for diabetes management, and potentially other age-related conditions.

## 2. Materials and Methods

### 2.1. Structural Design of the Portable Autofluorescence Detection System

**Figure 1:**
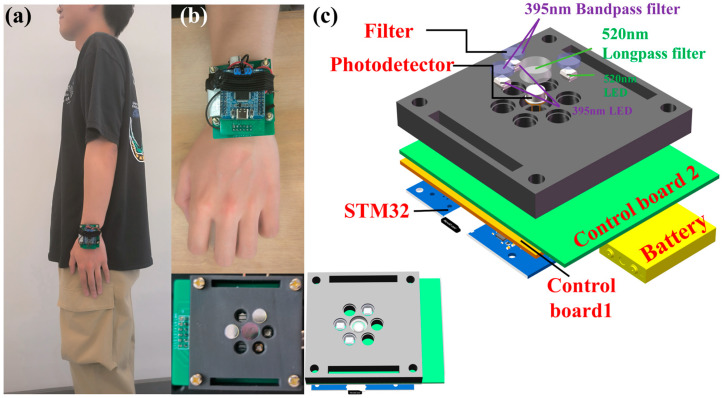
Structural design of the portable autofluorescence detection system. (**a**) Schematic of the device worn on the arm, with dimensions of 60 mm × 50 mm × 20 mm, secured with an elastic band. (**b**) Actual device displaying various modules. (**c**) Three-dimensional structural design illustrating the 395 nm excitation module with a bandpass filter emitting light onto the forearm to stimulate AGEs’ fluorescence; the emitted fluorescence is then received by the detector through a 520 nm long pass filter.

As depicted in Figure 1a, the device, designed for attachment to the wrist, has a length of 60.00 mm and a width of 50.00 mm, enabling portability and continuous, on-the-go monitoring. It employs a dual-LED configuration with an emission wavelength of 395 nm to elicit the characteristic fluorescence of AGEs. The emitted fluorescence is detected by an S1223 photodetector. To ensure the integrity of the signals and facilitate data processing, the device integrates a three-layered circuit board that controls the system, processes the signals, and manages data handling. The LEDs are encased within a black housing fabricated using 3D printing technology, which serves to prevent light leakage that could potentially interfere with the accuracy of detection. Additionally, filters are embedded within this housing to refine the wavelength specificity, as detailed in the structural representation in Figure 1c. This design exemplifies a seamless amalgamation of functionality and sophistication, crucial for the effective non-invasive monitoring of AGEs in clinical and everyday settings. Table 1 enumerates all of the critical components incorporated within the device, detailing their specifications, parameters, and individual prices. This facilitates the evaluation of the device’s operational capabilities and aids in budgetary planning.

### 2.2. Enhanced Control Module Design for Precision and Portability

**Figure 2:**
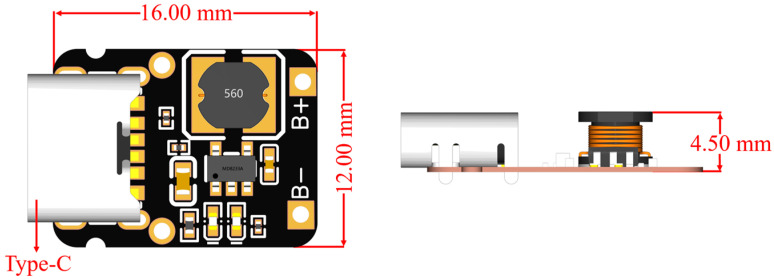
Compact integrated charging and discharging module dimensions: 16.00 mm × 12.00 mm × 2.60 mm.

At the heart of the device’s control apparatus lies an STM32 circuit board, strategically positioned on the uppermost layer with dimensions of 39.00 mm by 33.40 mm, featuring an STM32 microcontroller chip. The control of LED pulsation is governed by the I/O port voltage fluctuations on the STM32 circuit board, while the onboard analog-to-digital converter (ADC) of the STM32 reads the voltage output from the detection module for analog-to-digital conversion and data processing. To address the issue of insufficient output power from the STM32 microcontroller’s I/O ports, a dedicated transistor amplification circuit has been devised. A high-gain NPN transistor with a collector current of 3 A and a collector-emitter breakdown voltage of 30 V forms a common-emitter current amplification circuit with negative feedback, which effectively amplifies the nominal 10 mA current from the control board. By meticulously adjusting the transistor’s operating point and the negative feedback network, the 10 mA output from the STM32 chip is amplified to approximately 500 mA, which is sufficient to drive the LED lights, thus ensuring their stable and uniform irradiation. For enhanced portability, a compact charging and discharging module (as shown in Figure 2) is connected to a 3.7 V lithium battery. This module stabilizes the boost output to 5 V, supplying power to the STM32 circuit board and the entire system.

### 2.3. Optimized LED Emission Module for Enhanced Autofluorescence Excitation

The study by M. Koetsier et al. suggests that within the 355–405 nm wavelength range, the induced autofluorescence in skin shows no significant variance [25]. Based on these findings, our chosen excitation light source consists of two LED beads with a peak emission wavelength of 395 nm. These LEDs feature a 15 mm spectral half-width, a 60° emission angle, and a 3 W output power, and are soldered onto Control Board 2. Utilizing two 395 nm bandpass filters, extraneous wavelengths from the light source are eliminated, allowing the directed light to excite the autofluorescence of AGEs within the volunteer’s wrist skin. Additionally, an LED with a central emission wavelength of 520 nm serves as the calibration light source to mitigate the influence of varying skin types on fluorescence detection. By processing the fluorescence signal intensities under both wavelengths through algorithms, we can more accurately assess the AGEs content. The circuit design includes three unused LED positions, ensuring they do not interfere with the detection outcome. Control Board 2 measures 60.00 mm in length and 50.00 mm in width, featuring a central circular recess in its design.

### 2.4. Advanced Detection Module with Precision Filtering and Amplification Circuitry

The intricate design of the detection module is centralized within Control Board 1, a highly specialized spectral analysis circuit board with dimensions of 50.00 mm by 50.00 mm. The photodetector S1223 is expertly interfaced with Control Board 1, penetrating the circular recess of Control Board 2 to sit within a 3D-printed black enclosure. This strategic placement ensures the detector is optimally close to the volunteer’s skin for maximum signal capture. A dedicated 520 nm optical filter is adeptly utilized to discriminate against any extraneous light, selectively passing only the fluorescence emanating from AGEs proteins. The S1223 photodetector captures this filtered light, converting it into a measurable voltage signal. To bolster detection precision, Control Board 1 incorporates a bespoke circuit for filtering and signal amplification to ensure that even the most delicate fluorescent signals are effectively discerned and amplified. These processed signals are then routed through an output terminal to the STM32 circuit board, where they undergo meticulous analog-to-digital conversion and further processing for accurate AGEs quantification.

## 3. Results

### 3.1. Study on the Correlation between Glycated Hemoglobin and AGEs’ Fluorescence Intensity

**Figure 3:**
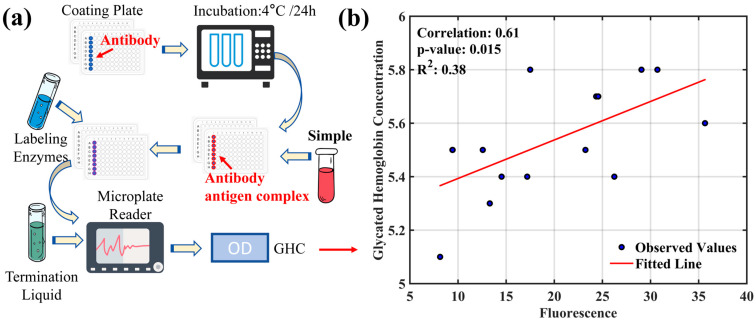
ELISA measurement and regression analysis of glycated hemoglobin and AGEs. (**a**) The ELISA process with coating plates, incubation, antibody-antigen complex formation, addition of labeling enzymes, microplate reading, and application of termination liquid. (**b**) The regression analysis demonstrating the relationship between glycated hemoglobin concentration and AGEs’ fluorescence, with a correlation coefficient of 0.61, a *p*-value of 0.015, and a coefficient of determination (R^2^) of 0.38.

Glycated hemoglobin (HbA1c) is a product of the binding of glucose in the blood with hemoglobin in red blood cells. It forms a stable compound through the covalent bonding of glucose with the N-terminal valine of the hemoglobin beta chain, and serves as an index of average blood plasma glucose levels over the preceding 8–12 weeks [26,27]. It is a gold standard for assessing glycemic control and gauging the risk of chronic complications in diabetes patients [28]. Notably, individual glucose levels are influenced by dietary habits, health status, and physical exercise load over time [29]. Furthermore, serum AGEs levels correlate with dietary intake, with high-protein or high-fat foods cooked at high temperatures being rich in dietary AGEs [30]. This section explores the correlation between HbA1c levels and AGEs’ fluorescence intensity in volunteers. Figure 3a illustrates the process of detecting glycated hemoglobin. To ensure experimental accuracy, blood samples from all volunteers were collected after fasting. The ELISA assay, utilizing a kit designed for blood glucose detection, provided an effective and precise means of quantifying glycated hemoglobin. The ELISA plates were coated with HbA1c-specific antibodies to capture the analyte effectively. The volunteers’ blood samples were then incubated in the plate wells at 4 °C for 24 h, allowing for the binding of HbA1c to the coated antibodies. After incubation, the wells were thoroughly washed to remove non-specifically bound proteins and potential interferences. Horseradish peroxidase (HRP)-conjugated secondary antibodies, which have a high affinity for HbA1c, were then added, forming stable immune complexes with the bound HbA1c during a secondary incubation. Additional washing removed any unbound enzyme-labeled secondary antibodies, preparing them for signal detection. TMB, a chromogenic substrate, underwent an oxidation reaction catalyzed by HRP, changing from colorless to blue, indicating the presence of HbA1c. The reaction was initiated by adding TMB to the system and halted at a predetermined time by a stop solution to prevent over-catalysis. This ensured the stability of the color change for reliable optical density (OD) measurements. The OD values were obtained by using a microplate reader, correlating with the HbA1c concentration in the samples. After an ELISA measurement, AGEs’ fluorescence intensity was measured on the volunteers’ wrists using a fluorescence detector, and the data were statistically fitted with the HbA1c levels determined by ELISA.

Figure 3b illustrates the correlation between HbA1c and AGEs’ fluorescence values obtained in this experiment, with a coefficient of determination (R^2^) of 0.38. This indicates that the changes in HbA1c levels account for 38% of the variation in AGEs’ fluorescence intensity, suggesting a moderate correlation between the two. However, the correlation is not strong, implying that other factors, in addition to HbA1c, may influence the formation and accumulation of AGEs.

### 3.2. Study on the Trends of AGEs’ Fluorescence Intensity and Blood Glucose Variation

To investigate the potential connection between the content of AGEs and real-time blood glucose levels, and to analyze their synchrony and correlation at different times of the day, we enlisted three volunteers to perform bi-daily tests of AGEs content at fixed times (8 a.m. and 8 p.m.). This testing continued for 5 days to capture any potential circadian rhythm fluctuations. In addition to measuring the fluorescence intensity of AGEs with a fluorescence detector during each test, the volunteers also used an Abbott blood glucose meter for finger-prick blood sampling to record immediate blood glucose levels.

**Figure 4:**
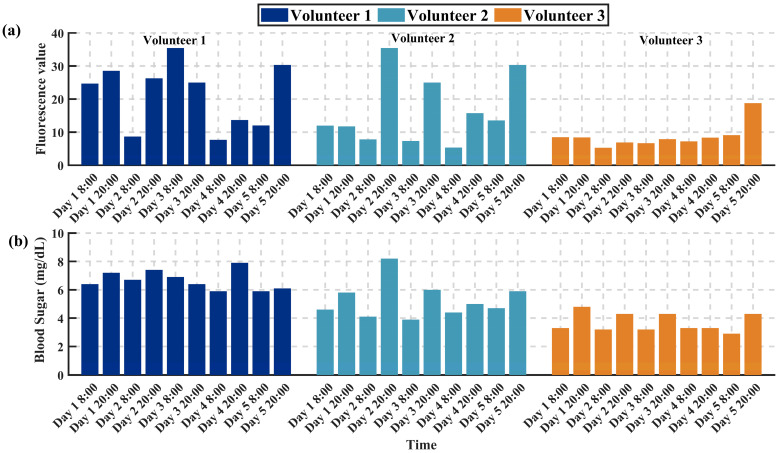
Investigation of AGEs content and real-time blood glucose levels over time. (**a**) Fluorescence intensity of AGEs content for Volunteers 1, 2, and 3 measured at different times. (**b**) Blood glucose levels of Volunteers 1, 2, and 3 measured at different times.

The experimental data displayed in Figure 4 indicate cosine similarities between the fluorescence intensity of AGEs and blood glucose levels for the three volunteers, which were 0.91, 0.93, and 0.95, respectively. This suggests a significant synchronous fluctuation relationship between AGEs’ fluorescence intensity and blood glucose levels during the observed time periods, implying a close association between AGEs formation and daily fluctuations in blood glucose levels, with a certain level of consistency across different individuals. However, while cosine similarity provides a quantifiable method for comparing similarities between the two, it does not directly infer causality. Additionally, we cannot overlook the potential differences among individuals. These differences may be caused by various factors, including but not limited to dietary habits, sleep quality, daily activity levels, and genetic factors. Therefore, future research should consider including more samples and exploring a wider range of potential influencing factors to further investigate how blood glucose levels affect AGEs formation, and how this influence is manifested through circadian rhythm changes.

### 3.3. Validation of a Multivariate Prediction Model Based on Analysis of Factors Influencing Advanced Glycation End-Product Content

**Figure 5:**
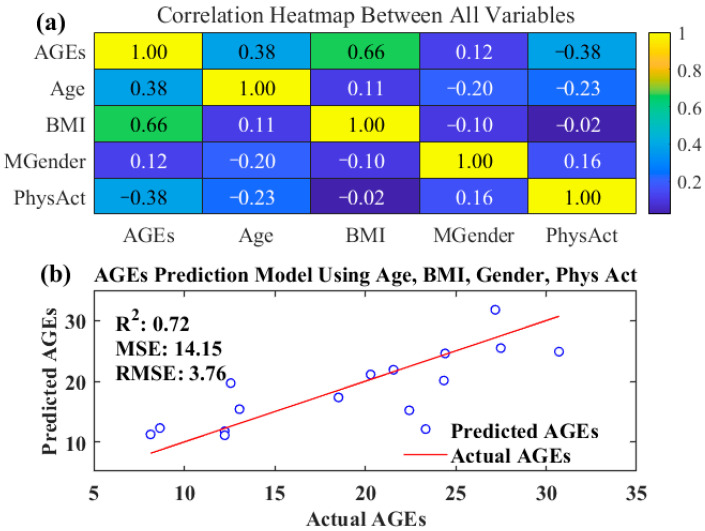
Analysis of influencing factors on AGEs content in human skin. (**a**) Correlation heatmap between AGEs, age, BMI, gender, and physical activity; (**b**) validation of AGEs prediction model using age, BMI, gender, and physical activity.

A multivariate prediction model validation based on the analysis of factors influencing the content of advanced glycation end-products (AGEs) was conducted. Fluorescence intensity of AGEs in the skin of 14 volunteers was measured using a fluorescence detector, and statistical analysis was performed on potential influencing factors such as age, body mass index (BMI), gender, and physical activity index (Phys Act) of the volunteers. By constructing a correlation heatmap, as shown in Figure 5a, we could visually observe the correlation between AGEs content and these variables.

The statistical analysis revealed a moderate positive correlation between AGEs content and volunteers’ age, with a correlation coefficient of 0.3824. This suggests that AGEs accumulation increases gradually with age, consistent with previous findings regarding the correlation between AGEs and the aging process [31,32]. Additionally, a strong positive correlation (correlation coefficient = 0.6627) was observed between AGEs content and BMI, indicating a significant association between obesity and AGEs formation and accumulation. Obese individuals may have higher levels of AGEs, possibly due to chronic low-grade inflammation, increased oxidative stress, and consumption of lipid-rich foods. The correlation between gender (Gender) and AGEs content was weaker (correlation coefficient = 0.1218), suggesting a minor influence of gender on AGEs formation and accumulation. However, this does not entirely rule out the potential role of gender in AGEs-related diseases, which may require larger sample sizes and further investigation.

Notably, AGEs content exhibited a negative correlation with physical activity (PhysAct) (correlation coefficient = −0.3822), implying that higher levels of physical activity may be associated with lower AGEs levels. This finding supports the potential benefits of a proactive lifestyle and exercise in slowing AGEs accumulation and improving health outcomes. Furthermore, a predictive model for individual AGEs content was established using AGEs, gender, and physical activity, which are highly correlated factors. Regression analysis of the predictive model’s results against the actual measured AGEs content further validated the association between these factors and AGEs content (see Figure 5b), yielding a coefficient of determination (R^2^) of 0.72. This high R^2^ indicates a good predictive capability of the model and underscores the significant predictive value of AGEs, gender, and physical activity for AGEs content.

## 4. Conclusions

This study designs and implements a portable AGEs detection device based on the spontaneous fluorescence of skin AGEs, which effectively measures the fluorescence intensity of skin AGEs. Experimental results indicate a certain degree of correlation between AGEs concentration and blood glucose levels, and through the validation of a multivariate predictive model, reveal the complex influencing factors of AGEs accumulation. The device offers real-time, non-invasive, and portable monitoring, making it suitable for large-scale screening and long-term monitoring. It provides a new tool for the early detection and prevention of chronic diseases such as diabetes, and holds promise for playing a crucial role in the early identification and risk assessment of chronic diseases like diabetes.

## 5. Discussion

The primary exogenous sources of advanced glycation end-products (AGEs) are derived from dietary intake, specifically from foods high in AGEs. Increased consumption of such foods is closely linked to elevated levels of AGEs in plasma and urine [33,34,35]. These high-AGEs foods, often processed at high temperatures through methods such as frying and grilling, typically contain high amounts of sugar. Excessive consumption can lead to increased blood glucose levels, establishing a connection between the intake of high-AGEs foods and higher blood glucose levels [36]. The affordability and convenience of the wearable devices used in this study enable us to expand our research into a broader population and implement long-term dietary tracking. This expansion will facilitate the collection of more comprehensive data, thereby enhancing the robustness of our findings.

Moreover, the research has revealed that levels of advanced glycation end-products (AGEs) are correlated with BMI, gender, and physical activity index, offering new perspectives for predicting AGEs content and blood glucose levels. Notably, BMI, which to some extent reflects food intake [37], suggests that excessive intake leads to the accumulation of AGEs. Therefore, a higher BMI may correlate with elevated AGEs levels due to increased consumption of foods rich in AGEs. However, it is crucial to thoroughly consider and account for the various biological and social differences that may underlie the observed variations in AGEs levels and their correlation with blood sugar. For instance, individuals with the same BMI might exhibit markedly different metabolic conditions. Recognizing these factors is essential. To improve the accuracy and reliability of our measurements, we will conduct rigorous dietary control experiments and monitoring. Initially, we will quantify AGEs in the diet and record the sugar content and calories in food to better understand the relationship between AGEs and blood sugar levels. Secondly, we plan to expand our research, which will help reduce variability and provide more robust data.

Additionally, understanding the biochemical pathways through which dietary AGEs influence blood glucose levels could provide further insights into metabolic health and disease prevention. Advanced glycation end-products (AGEs) have been shown to influence various biological processes. For instance, recent studies indicate that AGEs can regulate skin glycation by inhibiting specific transcription activators [38]. Additionally, the use of dual-channel fluorescence probes has enhanced the accuracy of detecting intracellular changes, which is crucial for understanding the relationship between AGEs and blood glucose levels [39]. Integrating these methods and insights into our future research will facilitate a clearer comprehension of the variability in the correlation between AGEs levels and blood glucose. This integration may enhance the predictive capabilities and clinical applications of our system. This line of research could lead to the development of targeted dietary recommendations that mitigate the impact of high-AGEs foods, potentially reducing the risk of diabetes and other related chronic conditions.

In future studies, we also plan to use our wearable devices for continuous monitoring and long-term tracking of AGEs and blood sugar levels. The miniaturization and wearability of our devices will enable us to conduct more extensive experiments and large-scale studies. This could enhance the accuracy and robustness of our findings on the relationship between AGEs and blood sugar levels. By integrating these strategies and methods, we aim to gain a clearer understanding of the variability in the correlation between AGEs levels and blood sugar, thereby enhancing the predictive capabilities and potential clinical applications of our system.

## Figures and Tables

**Table 1:** Key Components of the Portable Autofluorescence Detection System Used in This Study.

Device	Parameters	Cost
Excitation light	395 nm\3 W	0.69$
Calibration light source	520 nm\1 W	0.69$
Batteries	3.7 V\500 mAh	2.00$
Photodetector	0.45 A\W	9.00$
Excitation filter	10 mm\395 ± 10 nm	13.00$
Optical emission filter	10 mm\520 ± 10 nm	4.80$
Total		30.18$

## Data Availability

Data available upon request from the authors.
